# Comparative Assessment of Cytokines and Other Inflammatory Markers for the Early Diagnosis of Neonatal Sepsis–A Case Control Study

**DOI:** 10.1371/journal.pone.0068426

**Published:** 2013-07-15

**Authors:** Akila Prashant, Prashant Vishwanath, Praveen Kulkarni, Prashanth Sathya Narayana, Vijaykumar Gowdara, Suma M. Nataraj, Rashmi Nagaraj

**Affiliations:** 1 Department of Biochemistry, JSS Medical College, JSS Univeristy, Mysore, Karnataka, India; 2 Department of Community Medicine, JSS Medical College, JSS University, Mysore, Karanataka, India; 3 Department of Pediatrics, JSS Medical College, JSS University, Mysore, Karnataka, India; 4 Department of Microbiology, JSS Medical College, JSS University, Mysore, Karnataka, India; King’s College London School of Medicine, United Kingdom

## Abstract

**Objective:**

Cytokines (IL-6, IL-8 and TNF-α), sCD163, and C-reactive protein were serially measured in an attempt to identify a set of tests which can reliably confirm or refute the diagnosis of neonatal sepsis at an early stage.

**Methods:**

One hundred neonates suspected to have sepsis on clinical grounds and who met the inclusion criteria were enrolled for the study. Based on the positive or negative blood culture reports they were classified as infected (n = 50) and non-infected (n = 50) neonates respectively. Fifty healthy neonates without any signs of sepsis were also included in the study as control group. The initial blood sample was taken on day 0 (at the time of sepsis evaluation) and two further samples were taken on days 1 and 2 for monitoring the clinical progress and response to treatment. In the control group the cord blood and 48 hours venous sample was collected. Plasma CRP (ng/ml), IL-6 (pg/ml), IL-8 (pg/ml), TNF-α (ng/ml) and sCD163 (ng/ml) were determined by double antibody method Enzyme Linked Immunosorbent Assay in all the three blood samples.

**Results:**

The cut of levels for CRP at >19,689 ng/ml had a sensitivity of 68%, specificity of 92%, for IL-6 at >95.32 pg/ml had a sensitivity of 54%, specificity of 96%, for IL-8 at >70.86 pg/ml had a sensitivity of 78%, specificity of 70%, for sCD163 at >896.78 ng/ml had a sensitivity of 100%, specificity of 88% for the diagnosis of infection before antibiotics. TNF-α levels of >12.6 ng/ml showed 100% sensitivity and 72% specificity for the diagnosis of inflammation.

**Conclusion:**

The most powerful predictor to differentiate between the non-infected and infected neonates before antibiotics was sCD163. The most powerful indicator for evaluation of prognosis is IL-6. sCD163 can be used alone to screen for sepsis in neonates before the results of blood culture are received.

## Introduction

Bacterial sepsis is one of the major causes of neonatal morbidity and mortality [1]. Prevention and control of these infections are major challenges for neonatal intensive care units (NICUs). Rapid diagnosis, however, is problematic because the earliest signs of infection may be minimal and resemble those caused by various noninfective conditions [2]. Bacterial cultures are time-consuming and other laboratory tests are either not available for routine assay or lack sensitivity or specificity. In this situation, neonates with risk factors for infection or clinical suspicion of infection are empirically treated with antibiotics. On the other hand, the presence of multidrug-resistant organisms complicates the choice of antimicrobials. To avoid unnecessary treatment of noninfected neonates, an early, sensitive and specific laboratory test would be helpful to guide clinicians in neonatal units to decide whether or not to start antibiotics. Over the past few decades, some of the commonly used markers in clinical practice, especially leukocyte indexes and acute-phase reactants have been tried for early detection of neonatal sepsis.

The recent interest has shifted to chemokines, cytokines and interferons whose circulating levels may be different in healthy and infected subjects [3]. Although many of these trials have shown a positive correlation between these markers and infection, most were performed on newborns with wide range of birth weight and gestational age, involving only a small sample size, assessing only one or two markers at any one time and did not serially monitor the profile of these markers. Hence, the best single biochemical marker or combination of markers for the detection of neonatal sepsis has not yet been derived. In the review published on diagnostic markers for neonatal sepsis [4] it is recommended that there is a vast scope for searching newer diagnostic markers especially with cytokines either individually or in combination to detect neonatal sepsis.

Specific leucocyte surface antigens are known to be expressed in substantial quantities after inflammatory cells are activated by bacteria or their cellular products. Neutrophil CD11b and CD64 have been found to be promising markers for diagnosis of early and late onset sepsis [5]. However, there is growing interest in defining the potential diagnostic use of CD163 and its soluble form as novel markers of inflammatory disease activity [6].

Hence, in the present study we propose to do a comparative assessment of interleukin-6 (IL-6), interleukin-8 (IL-8), TNF-α and sCD163 with C-reactive protein (CRP) before antibiotics, for the early identification of neonatal sepsis and serial estimations at different time intervals to check the prognosis.

## Materials and Methods

### Source of Data

Jagadguru Sri Shivarathreeshwara Medical College ethical committee approval was taken before the commencement of the study.

Newborn infants with suspected sepsis on clinical grounds, admitted to the NICU of a tertiary care hospital, who met all inclusion criteria and whose parents signed an informed consent were enrolled in the study. Jagadguru Sri Shivarathreeshwara Medical college ethical committee has approved this study.

### Inclusion Criteria

Preterm and term neonates showing clinical signs suggestive of an early-onset (within 72 hours of birth), or late-onset (clinical deterioration after 3 days of age) infection were eligible for the study. The entry criteria for sepsis were physical signs of infection or rapid deterioration of respiratory and cardiovascular functions. Physical symptoms of infection were defined by the presence of at least three of the following: feeding intolerance; abdominal distension; temperature instability; disordered peripheral circulation (defined as paleness or peripheral cyanosis and mottled skin with a delayed capillary refill >3 seconds); lethargy or irritability; hepatosplenomegaly. Respiratory dysfunction were established by the presence of tachypnea (>70 breaths/min) or episodes of apnea and a >15% increase in the fraction of inspired oxygen (FiO_2_). Tachycardia (heart rate >190 beats/min) or bradycardia (heart rate <90 beats/min) and disordered peripheral circulation were regarded as symptoms of circulatory dysfunction.

### Exclusion Criteria

Newborn infants with congenital anomalies, chromosomal abnormalities or inborn errors of metabolism; confirmed intrauterine viral infection (toxoplasmosis, rubella, cytomegalovirus, syphilis and herpes); radiological findings consistent with necrotizing enterocolitis; who were already receiving parenteral antibiotic at the time of sepsis evaluation; and who had just undergone surgery were excluded from the study.

### Sample Size Calculation

The sensitivity of the cytokines and CRP observed in earlier studies ranged from 50% to 93%. The specificity of the measured cytokines and CRP ranged from 60% to 96%. A maximum sample size of 50 will yield approximately 25–30 cases satisfying the criteria of sensitivity. Hence 50 cases were included in each group.

### Classification of “Infective” Episodes

• ***Group 1 (Infected)*** **:** Neonates with clinical syndrome associated with a high probability of infection, positive findings on examination, imaging, or laboratory test and proven with positive bacteriological culture were included in this group.
• ***Group 2 (Non-infected)*** **:** Presence of at least 2 of 4 altered physiologic variables (temperature, heart rate, respiratory rate, leukocyte abnormalities) of which one was either abnormal temperature or leukocyte count with negative blood culture were included in this group. These neonates were classified as having systemic inflammatory response.
• ***Group 3 (control)*** **:** This group consisted of infants without any clinical evidence of sepsis (i.e., no sepsis evaluation undertaken; no antibiotics administered). For ethical reasons cord blood sample and one venous sample on the second day of birth was collected in this group of neonates.

At least 50 subjects were enrolled in each group. Antenatal history and anthropometric measurements such as birth weight, length, head circumference, of all the neonates were noted.

Immediately after the infants’ initial recruitment into the study, a relevant sepsis screening was performed. For the study purpose blood culture was performed using the approved guidelines of Clinical and Laboratory Standards Institute (CLSI) [7]. At the same time 2 ml of the blood was collected in plain evacuated blood tubes which was allowed to clot for 30 minutes and then centrifuged for 10 minutes at 1000×g. Serum was stored immediately at −80°C and then used for the estimation of CRP, IL-6, IL-8, TNF-α and sCD163. Fifty micro liter of the serum was used for each of these assays.

The initial sample was taken on day 0 (at the time of sepsis evaluation) and two further samples were taken on days 1 and 2 for monitoring the clinical progress and response to treatment. This schedule of blood sampling coincided with serial blood count and CRP measurements after the suspected episode of infection have been identified. Parenteral antibiotics were started immediately after the infection screen and first set of blood sampling had been performed.

Plasma CRP (ng/ml), IL-6 (pg/ml), IL-8 (pg/ml), TNF-α (ng/ml) and sCD163 (ng/ml) was determined by double antibody method Enzyme Linked Immunosorbent Assay [ELISA] using Quantikine ELISA kits from R&D system, *Inc*. as per the kit manufacturer’s instructions using automated ELISA reader and washer from Bio-rad laboratories, *Inc*. Performance characteristics of individual tests are as follows. Test for CRP had a sensitivity of 10 ng/ml and an intra-assay precision of 5–8.3% and inter-assay precision of 7.8–9.9%. IL-6 and IL-8 had a sensitivity of 0.92 pg/ml and 2.0 pg/ml respectively and an intra-assay and inter-assay precision of 3.4% and 5.2% for IL-6 and 6.3% and 8.7% for IL-8. TNF- α had a sensitivity to detect concentration of atleast 3.83 pg/ml and the test had an intra-assay precision of 6.9% and inter-assay precision of 7.4%. Similarly sCD163 had a sensitivity of 0.23 ng/ml and intra-assay precision of 3–6% and inter-assay precision of 5–8%. All the assay was run in duplicate with appropriate controls from R&D system, *Inc.*

### Statistical Methods

Arithmetic mean and standard deviation (SD) were estimated to assess the level of various parameters in the study. Differences in the levels of CRP, IL-6, IL-8, TNF-α and sCD163 between the defined groups were assessed using the Students ‘t’ test (if the comparison was between two groups) or repeated measure forward ANOVA (if the comparison was between more than 2 groups). Sensitivity, specificity, positive predictive value (PPV), negative predictive value (NPV), and positive or negative likelihood ratios (+ LR) for all the study parameters in predicting inflammation and infection at each point of follow up were calculated. Also, receiver operative characteristics (ROC) curves were constructed for each of the predictive variables and the areas under the ROC curves (AUC) were compared. Binary logistic regression analysis was used to determine; which of the above variable was an independent predictor for the diagnosis and outcome of neonatal sepsis. The analyses were facilitated with the use of the MedCalc 11.5.1.0 software packages. Differences were considered significant if the *P* value was <0.05.

## Results

The clinical characteristics of the study group are summarized in Table 1. A total of 150 neonates were included in the study with 50 belonging to each group. Table 2 shows the list of organisms grown in the blood culture of the infected group. The mean and standard deviation of all the study parameters in the infected, non-infected and control group at different time interval is shown in Table 3.

**Table 1:** Clinical characteristics of the study population.

Character	Infected group	Non-infected group	Control group
**Number of neonates**	50	50	50
**Birth weight (grams)**	2388±670**^b^**	2728±673	2920±439
**Length (cms)**	45.49±4.71^a^	46.81±4.06	47.49±3.29
**Head circumference (cms)**	32.02±2.42^a^	33.32±2.36	32.87±1.69
**Apgar scores:**			
**1 minute <7 (n = )**	20	17	3
**5 minute <7 (n = )**	9	7	0
**Male/Female**	33/17	35/15	27/23
**Early onset/Late onset**	41/9	46/4	–
**Term/Pre term**	29/21	39/11	43/7
**Vaginal/caesarean**	29/21	24/26	18/32

a p<0.05, ^b^p<0.001.

Pre term = born alive before 37 completed weeks of gestation.

**Table 2:** Pathogens isolated in blood cultures from 50 patients with bacteremia.

Pathogen	No of patients infected
Klebshiella Pneumonia	15
Staphylococcus aureus	7
group B Streptococcus	7
Acinobacter	7
Escherichia coli	6
Coagulase Negative Staphylococcus	5
Citrobacter	3

**Table 3:** The mean and standard deviation of the various study parameters in the infected, non-infected and control group at different time intervals.

Parameters	Blood sample	Infected groupMean ± SD	Non-infectedgroup Mean ± SD	Control groupMean ± SD	*P* value
**CRP ng/ml**	0	21942.48±5882.45^ b^	13082.18±6444.26^b^	43.61±15.09	<0.001
	24	22832.32±5660.81^ b^	13468.96±6330.21^ b^	671.15**±**549.16^ #^	<0.001
	48	23516.46±5684.48^ b^	13087.74±6447.66	–	<0.001
**P value**		0.312	0.942	0.0001	
**IL-6 pg/ml**	0	100.60±68.32^ b^	48.67±29.10^ b^	10.66±7.43	<0.001
	24	177.48±140.1^ b^	56.87±97.28	17.94**±**17.49^*^	<0.001
	48	185.54±157.31^ b*^	46.28±45.46	–	<0.001
**P value**		0.002	0.689	0.0027	
**IL-8 pg/ml**	0	122.28±72.60^ a^	78.36±69.78	76.88±82.37	= 0.003
	24	171.84±167.20^ b^	71.15±73.14	48.44**±**32.19^ *^	<0.001
	48	156.49±127.79^ b^	59.57±29.01	–	<0.001
**P value**		0.130	0.299	0.0038	
**TNF-α ng/ml**	0	25.11±14.06^ b^	24.78±15.49^ b^	11.64±9.66	<0.001
	24	22.70±13.18^ b^	31.91±25.65^ b^	11.17±7.66	<0.001
	48	37.63±27.03^ #^	31.54±23.20	–	0.236
**P value**		0.0001	0.190	0.0755	
**sCD163ng/ml**	0	1821.15±675.87^ b^	580.89±279.66	575.85±196.10	<0.001
	24	1790.09±610.71^ b^	669.04±343.14	637.83±170.60	<0.001
	48	1875.05±671.79^ b^	653.67±313.10	–	<0.001
**P value**		0.600	0.325	0.0808	

Horizontal: ^a^p<0.05, ^b^p<0.001, ^c^p<0.0001.

Vertical: * = p<0.05, ^$^p<0.001, ^#^p<0.0001.

All the neonates in the non-infected group had improved. None of the parameters showed any significant difference in the three blood samples collected at different intervals in the non-infected group. Out of the 50 patients in the infected group, 17 of them succumbed to the invading organism. The mean and standard deviation of all the study parameters in the survivors and non-survivors of the infected group at different time intervals is shown in Table 4.

**Table 4:** The mean and standard deviation of the various study parameters in the survivors and non-survivors of the infected group at different time intervals.

Parameters	Blood sample	Survivors Mean ± SD	Non-survivors Mean ± SD	*P* value
**CRP ng/ml**	0	20099.45±5203.09	25520.11±5587.75^b^	<0.001
	24	21028.30±5146.01	26334.23±5043.04^b^	<0.001
	48	21609.87±5521.27	27210.50±4007.12^b^	<0.001
**P value**		0.516	0.620	
**IL-6 pg/ml**	0	76.43±58.80	147.51±61.95^ b^	<0.001
	24	127.63±108.06	274.24±147.51^ b^	<0.001
	48	111.64±119.19	328.72±119.67^ b$^	<0.001
**P value**		0.103	0.001	
**IL-8 pg/ml**	0	107.86±65.52	150.28±79.34^ a^	0.049
	24	134.73±148.56	243.86±182.00^ a^	0.027
	48	124.92±114.66	217.66±133.19^ a^	0.017
**P value**		0.631	0.137	
**TNF- α ng/ml**	0	22.20±10.21	30.75±18.59^ a^	0.040
	24	22.01±8.98	24.06±19.18	0.607
	48	37.16±29.60^$^	38.54±22.06	0.870
**P value**		0.001	0.125	
**sCD163 ng/ml**	0	1744.72±673.50	1969.51±675.51	0.270
	24	1692.81±558.62	1978.93±678.73	0.118
	48	1767.29±560.45	2083.82±827.83	0.127
**P value**		0.878	0.884	

Horizontal: ^a^p<0.05, ^b^p<0.001, ^c^p<0.0001.

Vertical: * = p<0.05, ^$^p<0.001, ^#^p<0.0001.

**Figure 1:**
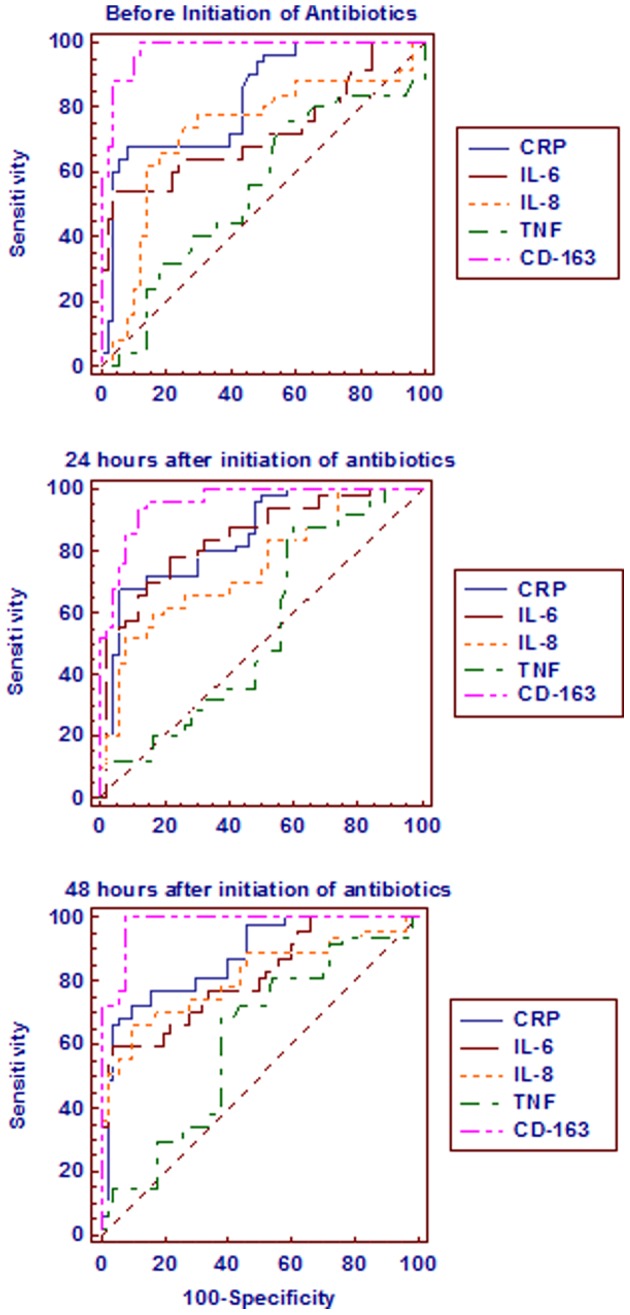
Comparison of the receiver operative curves of all the study parameters between the non-infected and infected neonates at different time intervals.

**Figure 2:**
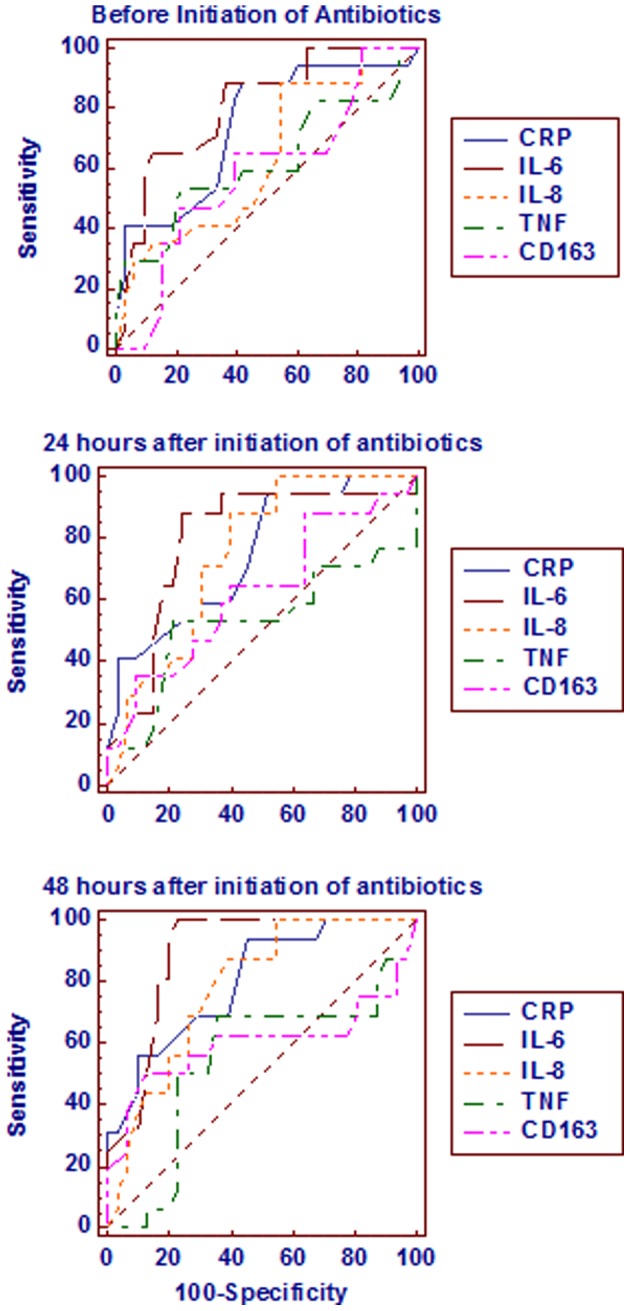
Comparison of the receiver operative curves of all the study parameters between the survivors and non-survivors of the infected group at different time intervals.

Cord blood CRP levels of >98 ng/ml showed 100% sensitivity and specificity and venous blood CRP levels of >2,399 ng/ml showed 98% sensitivity and specificity for the diagnosis of inflammation. Cord blood IL-6 levels of >22 pg/ml showed 84% sensitivity and 94% specificity and venous blood IL-6 levels of >16.35 pg/ml showed 84% sensitivity and 72% specificity for the diagnosis of inflammation. Cord blood IL-8 levels did not show any significant cut off levels. However, the venous blood IL-8 levels of >58.27 pg/ml showed 46% sensitivity and 84% specificity for the diagnosis of inflammation. Cord blood TNF-α levels of >9.7 ng/ml showed 100% sensitivity and 64% specificity and venous blood TNF-α levels of >12.6 ng/ml showed 100% sensitivity and 72% specificity for the diagnosis of inflammation. Cord blood and venous blood sCD163 levels did not show significant cut off levels for the diagnosis of inflammation. The sensitivity, specificity, positive predictive value (PPV), negative predictive value (NPV), positive or negative likelyhood ratio (LR) and area under the receiver operative curves (ROC) (AUC) of all the study parameters to differentiate between non-infected and infected neonates at different time intervals is shown in Table 5 and Figure 1 and to differentiate between the survivors and non-survivors of the infected neonates at different time intervals is shown in Table 6 and Figure 2.

**Table 5:** Predictive ability of the study parameters to differentiate between the non-infected and infected neonates at different time intervals.

Variable	Time interval	Cut off levels	Sensitivity	Specificity	+LR	-LR	PPV	NPV	AUC	*P* value
**CRP ng/ml**	0	>19,689^c^	68.00	92.00	8.50	0.35	89.5	74.2	0.825	0.0001
	24	>21,021^c^	68.00	94.00	11.33	0.34	91.9	74.6	0.848	0.0001
	48	>19,875^c^	72.34	90.00	7.23	0.31	87.2	77.6	0.873	0.0001
**IL-6 pg/ml**	0	>95.32^c^	54.00	96.00	13.50	0.48	93.1	67.6	0.723	0.0001
	24	>66.56^c^	78.00	78.00	3.55	0.28	78.0	78.0	0.844	0.0001
	48	>85.9^c^	59.57	96.00	14.89	0.42	93.3	71.6	0.811	0.0001
**IL-8 pg/ml**	0	>70.86^c^	78.00	70.00	2.60	0.31	72.2	76.1	0.725	0.0001
	24	>106.38^c^	52.00	92.00	6.50	0.52	86.7	65.7	0.748	0.0001
	48	>87.78^c^	65.96	90.00	6.60	0.38	86.1	73.8	0.812	0.0001
**TNF- α ng/ml**	0	>18.30	76.00	42.00	1.31	0.57	56.7	63.6	0.546	0.4296
	24	<24.51	88.00	40.00	1.47	0.30	59.5	76.9	0.552	0.3707
	48	>21.48	68.09	62.00	1.79	0.51	62.7	67.4	0.613	0.0489
**sCD163 ng/ml**	0	>896.78^c^	100.00	88.00	8.33	0.00	89.3	100.0	^#^0.977	0.0001
	24	>994^c^	96.00	86.00	6.86	0.05	87.3	95.6	0.956	0.0001
	48	>1044.6 c	100.00	92.00	12.50	0.00	92.2	100.0	^#^0.979	0.0001

c p<0.0001.

**Table 6:** Predictive ability of the study parameters to differentiate between the between the survivors and non-survivors of the infected group at different time intervals.

Variable	Time interval	Cut off levels	Sensitivity	Specificity	+LR	-LR	PPV	NPV	AUC	*P* value
**CRP ng/ml**	0	>22,645^a^	88.24	57.58	2.08	0.20	51.7	90.5	0.739	0.0023
	24	>21,693^a^	94.12	48.48	1.83	0.12	48.5	94.1	0.746	0.0016
	48	>23,973^c^	93.75	54.84	2.08	0.11	51.7	94.4	0.803	0.0001
**IL-6 pg/ml**	0	>127.5^c^	64.71	87.88	5.34	0.40	73.3	82.9	0.807	0.0001
	24	>150.19^c^	88.24	75.76	3.64	0.16	65.2	92.6	0.794	0.0001
	48	>109.14^c^	100.00	77.42	4.43	0.00	69.6	100.0	0.892	0.0001
**IL-8 pg/ml**	0	>84.4	88.24	45.45	1.62	0.26	45.5	88.2	0.634	0.1183
	24	>83.2^a^	88.24	60.61	2.24	0.19	53.6	90.9	0.750	0.0012
	48	>94.54^b^	87.50	61.29	2.26	0.20	53.8	90.5	0.784	0.0002
**TNF- α ng/ml**	0	>21.76	52.94	78.79	2.50	0.60	56.2	76.5	0.618	0.1722
	24	>21.48	52.94	78.79	2.50	0.60	56.2	76.5	0.529	0.7441
	48	>24.66	68.75	64.52	1.94	0.48	50.0	80.0	0.543	0.6308
**sCD163 ng/ml**	0	>2337	47.06	78.79	2.22	0.67	53.3	74.3	0.584	0.3359
	24	>2337	35.29	90.91	3.88	0.71	66.7	73.2	0.628	0.1347
	48	>2499	50.00	87.10	3.87	0.57	66.7	77.1	0.601	0.2596

a p<0.05, ^b^p<0.001, ^c^p<0.0001.

By performing binary logistic regression analysis, it was shown that the most powerful predictor to differentiate between the non-infected and infected neonates before antibiotics was sCD163. An increase of 1 ng/ml in sCD163 significantly increased the relative probability of infection by a factor of 1.008 (95% CI, 1.0032–1.013, *P = *0.002) (Table 7). The most powerful indicator for the prognosis of neonatal sepsis was IL-6 both at 24 and 48 hours (Table 8 and 9).

**Table 7:** Diagnostic predictors of neonatal sepsis before initiation of antibiotic therapy.

Study parameters before antibiotics	OR	95% C.I. for OR	*P* value
**CRP**	1.000	1.000	0.008
**IL-6**	1.003	0.983–1.024	0.743
**IL-8**	0.996	0.982–1.010	0.548
**TNF-α**	1.041	0.969–1.119	0.267
**sCD163**	1.008	1.003–1.013	0.002

Note: Binary logistic regression analysis.

C.I = Confidence interval; OR = Odds Ratio.

**Table 8:** Prognostic predictors of neonatal sepsis 24 hours after initiation of antibiotic therapy.

Study parameters at 24 hours after antibiotics	OR	95% C.I. for OR	*P* value
**CRP**	1.000	1.000–1.001	0.007
**IL-6**	1.007	1.001–1.013	0.016
**IL-8**	1.005	1.000–1.010	0.064
**TNF-α**	0.915	0.840–0.998	0.044
**sCD163**	1.002	1.000–1.003	0.038

Note: Binary logistic regression analysis.

C.I = Confidence interval; OR = Odds Ratio.

**Table 9:** Prognostic predictors of neonatal sepsis 48 hours after initiation of antibiotic therapy.

Study parameters at 48 hours after antibiotics	OR	95% C.I. for OR	*P* value
**CRP**	1.000	1.000	0.034
**IL-6**	1.009	1.001–1.018	0.026
**IL-8**	1.001	0.993–1.009	0.769
**TNF-α**	0.980	0.943–1.019	0.320
**sCD163**	1.001	1.000–1.002	0.185

Note: Binary logistic regression analysis.

C.I = Confidence interval; OR = Odds Ratio.

## Discussion

Neonatal sepsis is still a leading cause of death among newborns since symptoms are often wrongly interpreted due to their non-specific nature and late occurrence [8]. Cytokines (IL-6, IL-8 and TNF-α), sCD163, and a commonly used acute phase reactant protein (C reactive protein) were serially measured in this study in an attempt to identify a set of tests which can reliably confirm or refute the diagnosis of systemic infection at an early stage in an economical way.

This study was done for a period of two years and we have estimated CRP, IL-6, IL-8, TNF-α and sCD163 in the serum of the infected, non-infected and control neonates before antibiotics, at 24 hours after antibiotics and at 48 hours after antibiotics. CRP levels were increased in the infected as well as in the non-infected neonates at the time of diagnosis indicating that their levels increase in infection as well as in inflammation. CRP levels in the venous blood were also significantly increased when compared to the cord blood levels in the control neonates but the levels did not reach the levels in the non-infected or infected neonates. The non-survivors had a very high CRP levels when compared to the survivors in the infected group. There is a steady increase in the CRP levels in the infected neonates. However, in the non-infected neonates the levels showed a slight increase at 24 hours but started to fall by 48 hours. Our results show that CRP shows high specificity in confirming infection and high sensitivity in predicting mortality.

There are several studies which have used different cut off values for CRP ranging from 10–70 mg/L and have reported that the sensitivity of CRP for identifying serious bacterial infections (SBI) ranges from 63% to 95%, and specificity ranges from 40% to 91% [9]. One study looked at whether the time from onset of fever at which CRP was measured affected its sensitivity or specificity. They found no significant difference in sensitivity or specificity between CRP values collected before or after 12 hours from the onset of fever [10]. From the available research, there seem to be appropriate recommendations for the use of CRP in sepsis patients. High CRP does suggest the presence of SBI but must be used together with other investigations to inform clinical decision-making on a case-by-case basis.

IL-6 also showed a significant increase in the venous blood when compared to the cord blood of the control neonates. Like CRP, both the infected and non-infected neonates showed a significant increase of IL-6 when compared to the control neonates at all time intervals indicating that it is increased both in infection and inflammation. The non-survivors also showed a significant increase of IL-6 when compared to the survivors at all the three time intervals. The serial measurements of IL-6 also showed a similar pattern as that of CRP. There is an increase in the IL-6 levels in the infected group whereas the levels fall in the non-infected neonates after 24 hours of antibiotics. It is interesting to see that even in the infected neonates the levels of IL-6 fall after 24 hours of antibiotics in the survivors but continue to rise in the non-survivors. Our results show that IL-6 shows high specificity in confirming infection and is 100% sensitive to predict mortality at 48 hours after antibiotics.

Like our study previous studies have also established the value of cytokines to predict bacterial infection in the neonate with high sensitivity and specificity [11], [12]. The cut-off values obtained in this study following ROC analysis are in agreement with the cut-off values obtained by other investigators who measured IL-6 immediately postpartum. Messer et al. [13] found an optimal cut-off value of 100 pg/ml IL-6 in peripheral blood of premature and mature newborns collected postnatally. Lehrnbecher et al. [14] applied a cutoff of 150 pg/ml in cord blood samples of premature and mature newborns, albeit with a lower sensitivity. Singh et al. [15] reported generally lower IL-6 values in cord blood samples of infected newborns. Krueger M et al [16] reported an optimum cut off value of 80 pg/ml for IL-6 with a sensitivity of 87% and a specificity of 90%. Tasci Y et al. [17] reported that a cord blood interleukin-6 level >29 pg/ml was found to have 84% sensitivity and 72.5% specificity for predicting positive placental cultures and 74.1% sensitivity and 76.7% specificity for identifying cases of histologic chorioamnionitis. For predicting funisitis and positive newborn cord blood cultures a cord blood interleukin-6 level >39 pg/ml had 100% sensitivity and 81% specificity. In our study we have shown that IL-6 is a good indicator to evaluate the prognosis of neonates with sepsis. An increase of 1 ng/ml in IL-6 significantly increasee the relative probability of mortality by a factor of 1.007 (95% CI, 1.001–1.013, *P = *0.016) at 24 hours and by a factor of 1.009 (95% CI, 1.001–1.018, *P* = 0.026) at 48 hours after starting antibiotics.

IL-8 showed a significant decrease in the venous blood when compared to the cord blood in the controls. Unlike CRP and IL-6, IL-8 showed a significant increase only in the infected neonates when compared to the non-infected and control neonates indicating that it is increased only in infection and not in inflammation. The non-survivors also showed a significant increase of IL-8 when compared to the survivors at all the three time intervals. The serial measurements of IL-8 also showed a similar pattern as that of CRP and IL-6. There is an increase in the IL-8 levels in the infected group whereas the levels fall in the non-infected neonates after the initiation of antibiotics. Our results show that IL-8 has high specificity in confirming infection at 24 hours and at 48 hours and high sensitivity in predicting mortality at the same time interval.

Franz and co-workers had used IL-8 and CRP in order to avoid unnecessary antibiotics to be given and succeeded in reducing antibiotics by 40% compared with using CRP and immature to total neutrophil count I/T [18]. Krueger M et al. [16] reported a cut off value for IL-8 at 90 pg/ml, in the cord blood, with a sensitivity of 87% and specificity of 94%. Mehr SS et al. [19] had shown that the IL-6 and IL-8 concentrations were significantly increased in the definitely infected group when compared to the probable, uncertain and control group. They reported a cutoff value for IL-8 before antibiotics at >28 pg/ml with a sensitivity of 82% and a specificity of 81%.

TNF-α did not show a significant difference in the cord blood and venous blood sample of the controls. TNF-α showed a significant increase in both the infected and non-infected neonates when compared to the control neonates indicating that it is increased both in infection and inflammation. Their levels could not differentiate between the infected and non-infected neonates. The non-survivors showed a significant increase of TNF-α when compared to the survivors before the initiation of antibiotics. The serial measurements of TNF-α, showed steep increase in the infected neonates after 24 hours of antibiotics. There is an increase in the TNF-α level in the infected group whereas the levels fall in the non-infected neonates after 24 hours of antibiotics. Our results show that TNF-α has high sensitivity in diagnosing inflammation but could not differentiate between the infected and non-infected neonates, neither could its level predict mortality.

The combination of IL-6 and TNF-α is shown to be more sensitive than IL-6 or TNF-α alone in early-onset neonatal infection (98.5, 90 and 87.9%, respectively) [20]. Various combinations of IL-6, CRP and TNF-a can maintain sensitivities and negative predictive values above 90% for late-onset infection from presentation until 48 hours afterwards [5]. At the time of diagnosis, serum IL-1beta, IL-6, IL-8, and TNF-alpha levels of culture-proven sepsis were significantly higher than those of the control groups (P<.05). Also, at the time of diagnosis, IL-1beta, IL-6, IL-8, and TNF-alpha levels of culture-proven sepsis and culture-negative sepsis were significantly higher than levels at the seventh day after antibiotic treatment [21].

sCD163 did not show a significant difference in the cord blood and venous blood sample of the controls. It showed a significant increase in the infected neonates when compared to the non-infected and control neonates indicating that it is increased only in infection and not in inflammation. Though the levels of sCD163 were elevated in the non-survivors it was not statistically significant. The serial measurements of sCD163 did not show any significant difference in both the infected and non-infected neonates compared to their levels before antibiotics. However, a slight increase is seen in the infected neonates while a slight decrease is seen in the non-infected neonates 24 hours after antibiotics. Our results show that sCD163 has high sensitivity in diagnosing infection but not inflammation. An increase of 1 ng/ml in sCD163 significantly increases the relative probability of infection by a factor of 1.008 (95% CI, 1.0032–1.013, *P = *0.002). Hence, it can be used alone to screen for sepsis in neonates before the results of blood cultures are received. This will also avoid the need for combined markers which are more expensive and impractical in most of the clinical set-up. However, it could not predict mortality.

CD163 has a wide repertoire of functions [22]. The increased expression of CD163 is a part of the maturation of a monocyte to a phagocytic macrophage [23]. The expression of CD163 is upregulated by interleukin- 6 and glucocorticoids together with interleukin-10, and downregulated by LPS and interferon-*γ.* Membrane expression of CD163 on monocytes and neutrophilss could differentiate between patients with SIRS with sepsis and noninfectious SIRS [24]. High concentrations of soluble CD163 (sCD163) have been described in adult septic patients [25]. It has been shown experimentally with cultured monocytes that CD163 can be shed from the cell membrane after LPS stimulus [26]. We in our study have determined the cut off values for sCD163 at >896.78 ng/ml before antibiotics with a sensitivity of 100% and specificity of 88%, at >994 ng/ml 24 hours after antibiotics with a sensitivity of 96% and specificity of 86%, at >1044.6 ng/ml 48 hours after antibiotics with a sensitivity of 100%, specificity of 92%, to differentiate between infected and non-infected neonates.

The most powerful predictor to differentiate between the non-infected and infected neonates before antibiotics was sCD163. The most powerful indicator for evaluation of prognosis is IL-6. sCD163 can be used alone to screen for sepsis in neonates before the results of blood culture are received.

Further, it would be helpful to examine similar cytokine groups across studies to evaluate more carefully the actual impact of these substances on the neonate. Establishing normative values for infants at varying postmenstrual ages will be important for interpretation of cytokine values associated with specific outcomes.
